# Identification and structural elucidation of ozonation transformation products of estrone

**DOI:** 10.1186/1752-153X-7-74

**Published:** 2013-04-23

**Authors:** Pedro Alejandro Segura, Pearl Kaplan, Viviane Yargeau

**Affiliations:** 1 Department of Chemical Engineering, McGill University, 3610 University, Montreal, QC, H3A 2B2, Canada

**Keywords:** Transformation products, Differential analysis, Estrone, High-resolution mass spectrometry, Pharmaceuticals, Ozonation

## Abstract

**Background:**

Quantitative methods for the analysis of contaminants of emerging concern (CECs) are abundant in the scientific literature. However, there are few reports on systematic methods of identification and structural identification of transformation products. For this reason, a new method based on high-resolution mass spectrometry and differential analysis was developed in order to facilitate and accelerate the process of identification and structural elucidation of transformation products CECs. This method was applied to the study of ozonation transformation products (OTPs) of the natural hormone estrone (E1).

**Results:**

A control compare trend experiment consisting in the comparison of a control sample to several samples having been exposed to decreasing concentrations of O_3(aq)_ indicated that 593 peaks could be associated with OTPs. After applying various filters to remove background noise, sample contaminants and signal spikes, this data set was reduced to 16 candidate peaks. By inspection of the shape of these peaks, only two compounds OTP-276 (*m/z* 275.12930) and OTP-318 (*m/z* 317.14008) were considered as good candidates for further study. Multi-stage tandem mass spectrometry (MS^n^) experiments of SPE extracts of the ozonated samples of E1 and of a deuterium-labeled analogue (E1-d_4_) showed that OTP-276 and OTP-318 had carboxylic acid and hydroxyl functional groups, as previously reported for OTPs of other hormones. Structures for these two compounds were proposed based on their MS^n^ spectra.

**Conclusion:**

These results indicate that the method proposed is a systematic and rapid approach to study transformation products of CECs.

## Background

In the past 15 years, the presence and fate of contaminants of emerging concern (CECs) in the environment has been studied extensively. However, relatively less attention has been given to transformation products (TPs) [1]. TPs of CECs can be found in wastewater treatment plants or in the environment as a result of a multitude of abiotic and biotic factors (such as hydrolysis [2], photolysis [3], oxidation [4] and microbial metabolism [5]) acting on parent compounds. TPs are of environmental concern particularly if they are biologically active or resistant to biodegradation. Conservation of biological activity after transformation is plausible if the pharmacophore of the parent compound is preserved [6] and resistance to biodegradation can result after the formation of new functional groups capable of inhibiting microbial metabolism [7]. Recently, an international expert workshop concluded that the assessment of risks of metabolites and environmental transformation products of pharmaceutical and personal care products is among the top 20 key issues that need to be addressed by the research community [8].

For these reasons, researchers have started to investigate the formation of TPs and have proposed molecular structures based on mass spectrometry [9], especially TPs formed after ozonation [10] which is one of the most studied tertiary water treatments. Different approaches have been used in order to identify and elucidate the structure of ozonation transformation products (OTPs). Huber, et al. [11] used model compounds having the same reactive groups towards O_3_ as 17α-ethinylestradiol in order to simplify the identification process of the OTPs of this estrogen. Structural elucidation was performed with gas chromatography–mass spectrometry (GC-MS) and liquid chromatography-tandem mass spectrometry (LC-MS/MS) both at low resolution. Bila, et al. [12] compared the retention time and GC-MS spectra of the OTPs of 17β-estradiol with those of commercial pure standards that were predicted as potential OTPs, based on the molecular structure of the hormone and known O_3_ chemistry. The authors also used spectral libraries to identify potential matches. Radjenović, et al. [13], de Oliveira Pereira, et al. [14] and Larcher, et al. [15] employed LC coupled to a hybrid high-resolution mass spectrometer to separate, identify and assign structures to OTPs. Benner and Ternes [16] based the structural elucidation of the OTPs of propranolol on product ion fragmentation trees generated by LC coupled to multi-stage tandem mass spectrometry (MS^n^). In spite of these studies, there are still few publications in relation to the development of systematic methods of identification and structural elucidation of TPs. Kern, et al. [17] used a database to screen 1794 possible TPs of 52 organic contaminants in preconcentrated extracts of surface waters analyzed by high-resolution mass spectrometry (HRMS). The list was generated by computer prediction of potential microbial TPs as well as experimentally known TPs of 52 organic contaminants. Applying this method the authors were able to identify 19 TPs in the samples. Helbling, et al. [18] used LC-HRMS and data-dependent MS/MS experiments with target and non-target post acquisition processing and found five new TPs formed after microbial degradation of pharmaceuticals. Gómez-Ramos, et al. [19] also employed an accurate mass database of organic contaminants and their more abundant product ions. Their approach was successful to identify eight TPs of organic contaminants in effluent wastewater samples.

The primary objective of this study was to develop a systematic and faster method for the identification and structural elucidation of OTPs of CECs, using HRMS and differential analysis. This method was designed to automatize the identification process and reduce the time required for data interpretation and manual analysis of the large data files generated by HRMS. Identification of OTPs was based on a control compare trend experiment. This experiment consists in the comparison to a control of several samples in which a variable has been modified, in this case the concentration of O_3_. A similar approach using differential global profiling was reported previously, however it only compares two sets of samples: a control and a treated group [20]. The advantage of the control compare trend experiment for the identification of OTPs is the possibility of monitoring the signal of the studied CEC and its OTPs as a function of a variable known to affect the formation of OTPs. This approach facilitates the identification of the optimal concentration for the generation of OTPs and, by applying various data filters, the list of OTPs candidates can be narrowed down to a manageable number of peaks. Once the main OTPs of the target compound are identified, their structure can be elucidated by infusing preconcentrated ozonated samples directly into the mass spectrometer in order to perform MS^n^ experiments at high resolution.

To demonstrate the applicability of this approach, the hormone estrone (E1) was chosen as a model compound and its TPs after ozonation were investigated. As a naturally occurring steroid hormone, E1 is commonly excreted from the body in its active form and has been reported in municipal wastewater effluents at concentrations up to 96 ng L^-1^ in Canada [21]. Since wastewater treatment plants were not designed to remove this hormone and other CECs, E1 is continuously released via wastewater effluent into surface water bodies and levels as high as 112 ng L^-1^ have been reported in surface water in the USA [22]. These receiving streams may become sources of raw drinking water or indirectly contribute to contamination of other sources of drinking water sources, as suggested by the frequency of detection of E1 (in 79% of samples) in sources of drinking water in the United States [23]. E1 has been observed to be almost completely removed during wastewater disinfection using ozone [24]. However, bench-scale studies investigating the fate and transformation of E1 during ozonation have revealed the formation of OTPs [11,14].

## Materials and methods

### Ozonation of estrone samples

Estrone (E1) of high purity (99.0%) was purchased from Sigma-Aldrich. Ozone [O_3(g)_] was produced in-situ from O_2(g)_ using an Ozomax OZO4VTT generator (Granby, QC) and introduced in pure H_2_O using a gas-washing bottle in order to produce an aqueous ozone solution. The concentration of O_3(aq)_ in the solution was determined by means of the Beer-Lambert law by measuring the absorbance at 260 nm and a molar absorptivity value of 3300 M^-1^ cm^-1^[25]. Aliquots of this O_3(aq)_ stock solution (60–982 μL) were added to 9 mL of a standard solution of E1 in pure H_2_O (0.59 mg L^-1^, free pH with an initial value of 6.05 ± 0.05) using gas tight syringes according to specific E1:O_3_ molar ratios (1:0, 2:1, 1:1, 1:2, 1:5, 1:8). Therefore, applied ozone doses were in the range 0.05 mg L^-1^ to 0.84 mg L^-1^. According to Paraskeva, et al. [26], O_3_ doses of 4–10 mg L^-1^ are usually used in WWTPs for disinfection. However preliminary experiments showed that with the conditions used here, O_3_ doses of 1 mg L^-1^ were sufficient to remove E1 below the quantification limit (65 μg L^-1^). After addition of O_3(aq)_ the sample volume was brought to 10 mL by addition of pure water. Then samples were covered and stirred for 10 min.

### Liquid chromatography-high resolution mass spectrometry analysis of estrone

Initial and residual E1 concentration were measured by liquid chromatography-high resolution mass spectrometry (LC-HRMS) using an Accela LC system coupled to a hybrid mass spectrometer consisting in a linear ion trap interfaced to an orbital trap (LTQ Orbitrap XL), all manufactured by Thermo Fisher Scientific (Waltham, MA). LC separation was done using a stationary phase consisting of a Hypersil Gold column (50 × 2.1 mm, 1.9 μm) protected with a in-line Direct-Connection UHPLC 0.2 μm filter. All solvents used for mobile phase preparation were Optima LCMS grade and were manufactured by Fisher Scientific. The mobile phase was constituted of H_2_O (solvent A) and MeCN (solvent B). LC gradient was the following (%B): 0 min (10%), 1 min (10%), 6 min (80%), 6.1 min (97%), 7.6 min (97%). Re-equilibration time was 2.4 min. Ionization in the negative mode was chosen over ionization in the positive mode since previous studies [14,27] on the OTPs of estrogens have shown that the latter gave better results. Also preliminary experiments (results not shown) indicated better signals of OTPs of E1 with atmospheric pressure chemical ionization (APCI) than with electrospray ionization. For these reasons, the Ion Max API source was used with the APCI probe in the negative mode. APCI(−) source parameters were: vaporizer temperature = 400°C; sheath gas flow = 32 arbitrary units, auxiliary sheath gas flow = 5 arbitrary units; discharge current = 4 μA; capillary temperature = 250°C, capillary voltage = 30 V, tube lens = 70 V. Detection was performed in the orbital trap portion of the mass spectrometer at high resolution (*R*_FWHM_ = 41 000 at *m/z* 269). Scanning was performed from *m/z* 50 to 350. The full scan cutoff at *m/z* 350 was set based on the estimation that a change in the molecular mass > + 80 u was unlikely for an OTP of E1. For these settings mass accuracy was < 1.8 mmu for *m/z* 269.

### Identification of the main ozonation transformation products of estrone

Since a multitude of substances (solvents, air and glassware contaminants) can be ionized and detected in the full scan mode [28], it is necessary to use a software that can discriminate background signals from the OTPs of E1 present in the samples. To facilitate and accelerate the elucidation process a differential analysis software, Sieve 1.3 developed by Thermo Scientific, was employed. The control compare trend experiment in Sieve was selected in order to monitor the various peaks, which might be associated with TPs, as a function of O_3_ dosage. In this type of experiment a sample is designed as control to which others samples are compared and normalized. The sample E1:O_3_ 1:8 was selected here as control in the trend experiment knowing that any potential OTP of E1 would have, in this sample, an average intensity > 0 (otherwise the calculated ratio sample to control would return an error). Software settings were the following: frames = up to 1000 with signal threshold > 500 000; *m/z* start = 200; *m/z* stop = 350; *m/z* width = 0.02; Retention time start = 0 min; Retention time stop = 7.5 min; Retention time width = 1 min. Maximum number of frames and intensity threshold were set to limit the identification process to only major OTPs. Also the lower threshold for the *m/z* range of the frames was set in order to identify the OTPs most closely related to E1 and that may conserve some of its biological activity.

### Preconcentration and purification

In order to obtain higher amounts of the suspect OTPs of E1, the experiments described in *Ozonation of estrone samples* section were repeated using 250 mL rather than 10 mL. A semi-batch reactor was employed to produce the O_3(aq)_ solution [29]. The concentration of E1 was also increased to 1.3 mg L^-1^, corresponding to its solubility limit in H_2_O, 1.30 ± 0.08 mg L^-1^[30]. In order to obtain a maximum amount of the OTPs the E1:O_3_ ratio was set to 1:10. Ozonated samples were preconcentrated using solid-phase extraction (SPE) based on a method described previously [31]. Briefly, since it has been reported that many of the OTPs of E1 have carboxylic acid functions [11,14], ozonated samples were acidified to pH 2.1 with HCl 1 M in order to increase their SPE recovery with Waters Oasis HLB cartridges (6 mL, 150 mg). The cartridges were conditioned with 2 × 2.5 mL MeOH and 2 × 3 mL acidified H_2_O with HCl to pH 2.0. Samples were loaded into the cartridges in a Supelco 12-position manifold (St. Louis, MO) at flow rate of 1–4 mL min^-1^. After loading, cartridges were rinsed with 5 mL of H_2_O to wash out any salts and vacuum-dried at 0.68 bar for 5 min. Cartridges were eluted with 2 × 1.25 mL of MeOH and 2 × 1.25 mL of MeCN. Extracts were then evaporated to dryness using a Savant SPD 131DDA Speed Vac Concentrator connected to a RVT4104 refrigerated vapor trap, both manufactured by Thermo Fisher Scientific (Waltham, MA). Finally samples were reconstituted in 50% H_2_O-50% MeCN (v/v) and passed through Millipore Millex-LG syringe filters (filter diameter 4 mm, pore size 0.22 μm) before direct infusion into the mass spectrometer to perform MS^n^ experiments.

### Structural elucidation and confirmation of the main ozonation transformation products of estrone

Once the main OTPs of E1 were identified, aliquots of the preconcentrated ozonated samples containing the OTPs of interest were introduced in the LC-HRMS system by infusion using a glass syringe. The precursor ions of the OTPs of E1 were then isolated and multiple MS^n^ (*n*=2-4) experiments were performed on the product ions using collision-induced dissociation (CID) and higher energy collision-induced dissociation (HCD). The mass-to-charge ratio (*m/z*) of the product ions was measured at high resolving power measured at full width at half-maximum (*R*_*FWHM*_ ≈ 190 000 at *m/z* 231). For these settings mass accuracy was < 0.9 mmu for *m/z* 231. Elemental formulas were generated from the exact masses of only the frames that were identified as potential OTPs using the differential analysis software and using the “Elemental composition” function in QualBrowser program of Xcalibur 2.1. The elements in use were: C (0 to 18 atoms), O (0–10 atoms) and H (0–40 atoms). Mass tolerance was set to 10 mmu. Ring and double-bond equivalents (RDBE) value was set to −0.5 to 20 while charge was set to −1. Structures were proposed based on known fragmentation pathways for negative even-electron ions [32] as well as basic principles of the stability of the product ions and the neutral products [33].

**Figure 1:**
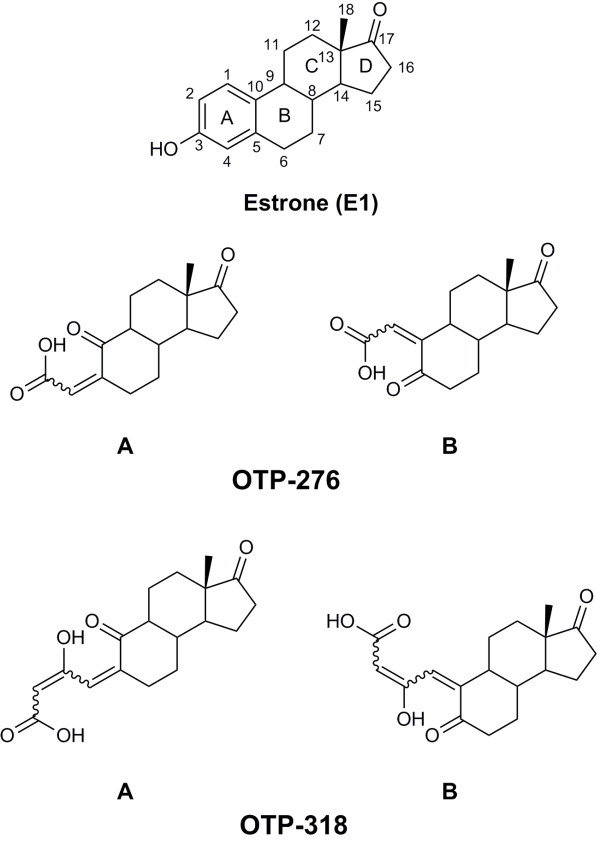
**Molecular structure of estrone (E1) showing the standard C atom numbering and two possible structures for each of the identified OTPs.** OTP-276B was not observed, while OTP-318B was 10 times less abundant than OTP-318A. Wavy bonds indicate that the exact stereochemistry is unknown.

In order to confirm the structure of the identified OTPs of E1, a deuterated E1 standard, estrone-2,4,16,16-d_4_ (99%) was bought from CDN isotopes (Point-Claire, QC). The E1-d_4_ standard used had two deuterium atoms (^2^H, but for simplicity D will be used instead) in the aromatic ring, in C-2 and C-4, and two geminal D atoms in C-16 (Figure 1). Semi-batch ozonation experiments, as described in section Preconcentration and purification, were performed with this standard to generate the same OTPs (but with different mass) in order to confirm the sites on the E1 molecule where reaction with O_3_ took place and propose structures of the MS^n^ product ions of the identified OTPs of E1.

## Results and discussion

### Identification of estrone ozonation transformation products by differential analysis

Preliminary experiments with the acquisition files showed that identification of OTPs of E1 based on present knowledge of O_3_ chemistry and previously reported OTPs was partially successful (only one compound was found, with a precursor ion at *m/z* = 275 which will be discussed later). For this reason, a control compare trend experiment, followed by differential analysis with the Sieve software was performed. Sieve found 593 unique “frames” in the samples. In this software, a frame represents the coordinates of a peak in a three-dimensional space that allows the identification of substances in the HRMS acquisition files. The coordinates of these frames are: retention time, *m/z* value and intensity. However, not all of these frames represent an OTP of E1. Many frames correspond to background signals and laboratory contaminants that are detected by the mass spectrometer. In order to eliminate the frames that are not relevant to the identification of OTPs of E1, the frames table filter function available in Sieve was used. This feature allows to set several rules that the frames must follow in order to qualify as a potential OTP. The rules are based on parameters such as minimum or maximum *m/z* value, retention time (t_R_) range, average frame intensity, normalized average frame intensity to control (samples E1:O_3_ 1:8), etc. We applied a set of three rules to identify the frames most probably corresponding to OTPs of E1: i) The normalized average intensity of a frame in the samples E1:O_3_ 1:0 must be < 0.05; ii) Normalized average intensity of a frame in samples E1:O_3_ 1:1 and 1:5 > 0.05; and iii) The average intensity of the frame in the samples E1:O_3_1:8 must be > 5000. The first rule ensured that frames present in the sample E1:O_3_ 1:0, which was not ozonated, had very low intensity compared to the intensity in the sample E1:O_3_ 1:8, which had the largest dose of O_3_. Normally a frame corresponding to an OTP should not be observed in the sample E1:O_3_ 1:0 but since some low carryover was observed between samples, residual ultra trace amounts were often detected in all the samples. High carryover could be problematic as it can mask significant differences between the samples. Therefore it was kept to a minimum by applying syringe washes and using blanks between samples. The second rule guaranteed that the filtered frames are not associated to random signal spikes since they have to be present in both samples E1:O_3_ 1:0 and 1:5. Therefore their normalized average intensity has to be different than zero. The normalized value of 0.05 was found to be optimal to eliminate most of frames containing peaks with unacceptable shape. The last rule ensured that only frames of relevant intensity are kept for further analysis since frames an average intensity of < 5000 were not considered as main OTPs. This rule also helped selecting frames with intensity sufficiently high to allow MS^n^ experiments. The filtered frame results are summarized in Table 1. It can be noted that only 16 out of the 593 initial frames were conserved and interestingly only two (frame #9 and #78) were among the 100 most intense frames. Visual examination of the reconstructed ion chromatograms showed that only these two frames (#9 and #78, eluting at t_R_= 3.51 and 3.32 min, respectively) had acceptable peak shape. The others had peaks with retention times that were not consistent across all the samples or had low signal-to-noise ratios. Looking at the retention times of the frames after the filter was applied, it can be seen that many of the frames identified by the differential analysis software as OTPs eluted by the end of the chromatographic run (t_R_ > 7.0 min). Inspection of these frames showed that they consisted mostly of intense noise with signal spikes and is unlikely that they were related to E1 (it is not clear, however, why these frames were not eliminated by the filters). Lower retention times than the parent compound are consistent with OTPs which by the addition of O and loss of C atoms tend to be more hydrophilic than the parent compound (t_R_ of E1 = 4.87 min) and thus less retained in reversed-phase columns. Therefore only frames #9 and #78 were kept for further investigation.

**Table 1:** Characteristics of the frames retained for further analysis by applying the filter option

**Frame number **^**a**^	***m/z***	**Retention time (min)**	**Normalized average intensities **^**b**^			**Average intensity in sample 1:8**
			**Sample 1:0**	**Sample 1:1**	**Sample 1:5**	
9	275.12930	3.51	0.009	0.062	0.614	22664
78	317.14008	3.32	0.022	0.075	0.914	5323
200	212.09456	7.38	0.050	0.486	0.863	8552
225	224.06932	7.49	0.026	0.442	0.739	6080
241	202.07396	7.33	0.049	0.483	0.926	11688
267	224.09468	7.44	0.036	0.456	0.916	7829
282	228.08983	7.40	0.039	0.297	0.694	6061
294	226.07419	7.46	0.000	0.456	0.943	6511
333	226.08574	7.40	0.000	0.417	0.652	6489
344	229.08455	7.41	0.049	0.334	0.540	5285
357	212.05852	7.46	0.041	0.466	0.784	5656
358	201.07838	7.45	0.041	0.477	0.869	7736
375	215.06937	7.46	0.044	0.451	0.566	5559
418	250.14478	4.29	0.038	0.071	0.239	9056
419	250.14525	3.70	0.027	0.086	0.303	8340
574	250.14519	2.99	0.038	0.051	0.240	8590

^a^ Frame number is assigned sequentially by the software according to absolute intensity in all samples. Therefore frame #1 represents the most intense frame found across all samples. ^b^ Average intensity of each frame was normalized to its average intensity in the samples to the control (sample 1:8).

The elemental compositions of the OTPs present in frame #9 and #78, presented in Table 2, were determined by QualBrowser according to the parameters mentioned in the *Identification of the main ozonation transformation products of estrone* section. Since these compounds are a result of the oxidation of E1, which has an elemental composition of C_18_H_22_O_2_, the number of elements and their constraints used for generating formulas with the Elemental Composition function in QualBrowser significantly reduced the number of possible matches. Given that after ozonolysis the number of C atoms in the OTPs is not expected to increase, the maximum number of C atoms was set to 18 (minimum was always set to 0). As for O, a value higher than 10 is not expected given that the number of reaction sites is limited (phenol function in ring A and ketone at C-17, see Figure 1). Finally for H, a maximum value of 40 was set as ring cleavage caused by O_3_ could lead to the formation of new functional groups containing H such as -COOH, -CHO and -OH [34,35]. Also in order to reduce the number of possible matches, the nitrogen rule and a mass tolerance of 10 mmu were employed. The software returned only one match for the composition of the OTP in frame #9, C_16_H_19_O_4_ (C_16_H_20_O_4_ for the neutral molecule) with a Δmmu=0.418. This composition implies that the reaction of E1 with O_3_ caused the loss of 2 C atoms and 2 H atoms as well as the addition of 2 O atoms. As for the OTP in frame #78, two elemental compositions were suggested. The most likely composition was C_18_H_21_O_5_ (addition of 3 O atoms with respect to E1) due to its RDBE value of 8.5 (same as that of E1). The other proposed composition, C_11_H_25_O_10_ (RDBE=−0.5), was a fully saturated compound that is a very unlikely possibility given that the pseudo-molecular ion of E1 has a RDBE=8.5 (four rings plus four π bonds, the 0.5 value is due to the loss of a H atom in the [M-H]^-^ ion).

**Table 2:** Elemental composition of the two potential OBPs identified by the differential analysis software compared to that of the parent compound E1

**Frame number**	***m/z***	**Elemental composition**	**RDBE**	**Δ mmu**
(E1)	269.15537	C_18_H_21_O_2_	8.5	0.667
9	275.12930	C_16_H_19_O_4_	7.5	0.418
78	317.14008	C_18_H_21_O_5_	8.5	0.633
		C_11_H_25_O_10_	−0.5	−5.240

RDBE: Ring and double bond equivalents. Δmmu: absolute mass accuracy in milimass units.

The results showed that the differential analysis method significantly reduces the time of data analysis and interpretation. Background noise, sample contaminants and signal spikes were thus mostly eliminated from the files by using a control and series of samples with decreasing concentration of O_3_ as well as a set of rules to filter the results. Therefore instead of performing manual analysis (elemental composition, acceptable peak shape) of 593 potential OTPs, this method reduced the data set to 16 compounds. While this method still suffers from an abundance of false positive hits (only 12.5% of the potential OTPs were good candidates) and may also ignore minor OTPs, it proved to be a rapid and simple way to identify the two main OTPs of E1 formed during the ozonation conditions described in the *Ozonation of estrone samples* section.

### Structural elucidation of OTPs

Preconcentration of ozonated samples was necessary in order to gain more information about the molecular structure of the identified OTPs by performing MS^n^ experiments. If concentrations of OTPs would have been sufficiently high in the ozonated samples, data-dependent experiments could have been performed to obtain additional structural information at the identification stage. However, in this study a SPE step was necessary in order to obtain signal intensities high enough for reliable MS^2n^ experiments. Injection of the SPE extract of the E1 ozonated solutions showed intense chromatographic peaks eluting at t_R_ = 3.52 min and 3.40 min with mass spectra showing base peaks at *m/z* 275.1 and *m/z* 317.1, respectively. These results confirmed that the OTP present in frame #9 (called OTP-276 from now on) and frame #78 (called OTP-318) were successfully preconcentrated by SPE. Interestingly, two other peaks were observed with t_R_=3.64 and t_R_=3.73 min at *m/z* 317.14 ± 0.01, which may imply that isomers of the compound were also formed (Additional file 1: Figure S1). Direct infusion of these extracts into the mass spectrometer allowed performing multi-stage tandem mass spectrometry (MS^n^) experiments with these ions. It is worth mentioning that the structural elucidation stage of the present method can be further simplified by using software capable of predicting collision-induced product ions [36]. This approach was unfortunately not possible here since the software available to us was not able to predict product ions of even-electron negative ions.

### Structural elucidation of OTP-276 by HRMS^n^ experiments

Ozone is a very selective oxidant towards electron-rich groups [37] and the E1 molecule has only two of such groups, the aromatic ring (ring A) and the ketone function at C-17 (Figure 1). Of these two, the most likely site of reaction is the aromatic ring since it has higher electron density, especially in the *ortho* and *para* positions to the electron-donating hydroxyl group at C-3 [35]. As determined in the previous section, the elemental composition of OTP-276 is C_16_H_19_O_4_, which indicates the loss of 2 C atoms and 2 H atoms as well as the addition of 2 O atoms with respect to E1. Fragmentation of the pseudo-molecular ion of OTP-276 (*m/z* 275) by collision-induced dissociation (CID) with 30 normalized collision energy (NCE) in the linear ion trap portion of the mass spectrometer yielded only one main first generation (MS^2^) product ion, *m/z* 231.13989 with the elemental composition C_15_H_19_O_2_ (Δmmu=0.837). The loss of 44 u with respect the precursor ion indicates the loss of CO_2_ by heterolytic cleavage. Decarboxylation is a characteristic fragmentation pathway for even-electron negative ions of carboxylic acids [32,38]. Therefore a carboxylic acid functional group must be present in the structure of OTP-276. This functional group could have been formed after ring cleavage and loss of two aromatic C atoms of E1 to form a ketone group on C-10 and a carboxylic acid on C-3 (OTP-276A, in Figure 1) after ozonolysis. This structure was previously suggested by de Oliveira Pereira, et al. [14]. It was explained by a two-step O_3_ attack, the first forming a molozonide intermediate yielding an aldehyde and a carboxilyc acid on C-2 and C-3 respectively, and the second forming a new molozonide intermediate that decomposes to form OTP-276 [14]. When performing a full scan (MS^1^) experiment on the SPE extract of ozonated E1-d_4,_ the formation of an isomer of this structure having ketone group on C-5 and the carboxylic group on C-2 (OTP-276B, Figure 1) with a formula C_16_H_17_D_2_O_4_^-^ was not observed. The ion at *m/z* 278.14758 (C_16_H_16_D_3_O_4_^-^, Δmmu=0.964) was observed instead; indicating that one of the aromatic D atoms was still present in the molecular structure. This experiment thus reinforces the validity of the proposed structure for (OTP-276A, Figure 1).

**Figure 2:**
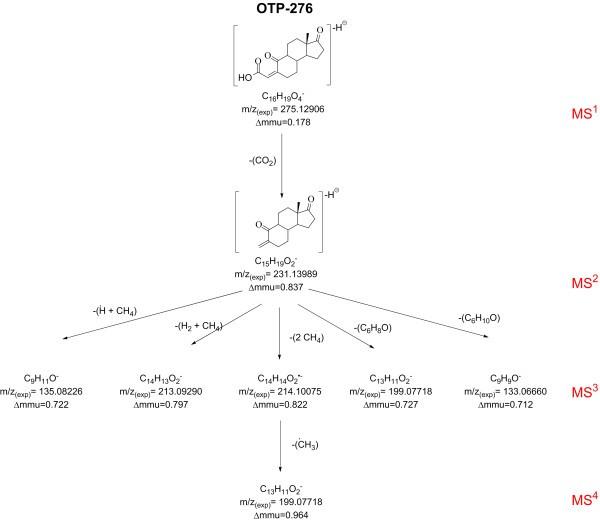
**Fragmentation tree of OTP-276 showing the most probable elemental composition of the main MS**^**n **^**product ions.**

Following these results, MS^n^ (*n*=3-4) experiments were performed with the precursor ion at *m/z* 275 in order to further confirm the proposed molecular structure of OTP-276. The fragmentation tree of *m/z* 275 showing the formulas of the observed MS^n^ product ions can be seen in Figure 2. The presence of the second generation (MS^3^) product ion at *m/z* 214 is particularly puzzling since it does not follow the nitrogen rule. Therefore this ion must be odd-electron, meaning that *m/z* 214 violates the even-electron rule which states that even-electron ions do not lose a radical to form an odd-electron ion [39]. However, such deviations of the even-electron rule have been previously reported after CID of even-electron ions [38,40]. The loss of 17 u was thus interpreted as the loss of H^•^ + CH_4_. The minor MS^3^ product ions observed in the full scan CID spectrum of *m/z* 231 at *m/z* 213.09290 (C_14_H_13_O_2_^-^, Δmmu=0.797), *m/z* 199.07718 (C_13_H_11_O_2_^-^, Δmmu=0.727), *m/z* 135.0826 (C_9_H_11_O^-^, Δmmu=0.722) and *m/z* 133.06660 (C_9_H_9_O^-^, Δmmu=0.712) indicate neutral losses of CH_6_, C_2_H_8_, C_6_H_8_O and C_6_H_10_O, respectively. Since the loss H_2_ and CH_4_ is a common pattern of fragmentation for negative even-electron ions [32], the neutral loss of CH_6_ was interpreted as the combined loss H_2_ + CH_4_, and that of C_2_H_8_ as 2CH_4_. Losses of CH_4_ can be explained by the dissociation in stepwise elimination mechanisms [32] of the methyl group from C-13 (the C atom numbering of E1 is preserved to facilitate the identification of the fragmentation sites) and the methylene group from C-5. However, the precise mechanism that could yield H_2_ and CH_4_ losses from *m/z* 231 remains unclear. Additionally, the formation of ions at *m/z* 135 and *m/z* 133 is also difficult to elucidate, however they are likely the consequence of stepwise elimination reactions and internal nucleophilic displacements [32]. The elemental compositions of the second generation product ions formed during the MS^3^ experiments were confirmed using the E1-d_4_ ozonated SPE extract (Additional file 1: Figure S2). The results of these experiments showed that a D atom was lost during the fragmentation of *m/z* 234.15759 (C_15_H_16_D_3_O_2_^-^, Δmmu=−0.293) to form *m/z* 201.08897 (C_13_H_9_D_2_O_2_^-^, Δmmu=−0.246). This D atom was most likely located in the methylene group on C-5. The third generation (MS^4^) product ion scan experiment (*m/z* 275 → *m/z* 231 → *m/z* 214 *↗ m/z* 50–300) with NCE=50 resulted in the presence of only one ion at *m/z* 199.07631. This third generation product ion was likely formed by dissociation of ^•^CH_3_.

In summary, MS^n^ experiments data showed that only the MS^2^ product ion at *m/z* 231 clearly demonstrates the presence of a carboxylic acid group in the OTP-276 structure. The ion at *m/z* 199 observed in the MS^3^ and third generation (MS^4^) product ion scans could have suggested the presence of a double bond between C-4 and C-5 in OTP-276, however the cleavage of this bond to yield the CH_4_ or (^•^CH_3_) neutral losses observed was not easily explained by known mechanisms. The rest of the neutral loses, albeit characteristic of negative even-electron ions, did not suggest the presence of other functional groups on the molecule. Product ions generated in the MS^3^ to MS^4^ experiments pointed towards a partial preservation of the ring structure of E1 and the observed losses (H_2_, CH_4_) were thus justified by the formation of resonance-stabilized ring structures.

### Structural elucidation of OTP-318 by HRMS^n^ experiments

Fragmentation of the precursor ion at *m/z* 317 resulted in the formation of a MS^2^ product ion at *m/z* 273.14960. The neutral loss (44 u) corresponds to CO_2_, which as in the case of OTP-276, indicates the presence of a carboxylic acid group in the molecular structure of OTP-318. Initially it was thought that the structure of OTP-318 was 1,2,4-trihydroxyestrone (hydroxylation at C-1, C-2 and C-4 of E1). This structure was rejected once it was observed that a CO_2_ loss was difficult to originate from such a compound. The carboxylic group in OTP-318 was most likely formed after the cleavage of the aromatic ring of E1 by O_3_. This was confirmed by the full scan spectrum of the ozonated extract of E1-d_4_ (Additional file 1: Figure S3), which yielded an abundant ion at *m/z* 321.16456 (C_16_H_17_D_4_O_5_^-^, Δmmu=0.006). Since the deuterated OTP conserved all its D atoms after ozonolysis, the O_3_ attack must have occurred at C-1 and C-10 rather than at C-4 and C-5, which would result in the loss the D atom at C-4 (OTP-318B, Figure 1). Interestingly the formation of an OTP-318 isomer resulting from the O_3_ attack at C-4 and C-5 (C_18_H_18_D_3_O_5_^-^, Δmmu=−0.217) was also observed in the mass spectrum at *m/z* 320.15806, but its signal was less intense, about 10 times less abundant than *m/z* 321.

MS^3^ and MS^4^ experiments were performed on the most abundant ions in order to gain more information about the structure of OTP-318. The MS^3^ product ion scan (*m/z* 317 → *m/z* 273 *↗ m/z* 75–350) showed the presence of five peaks with abundance > 10%. These were: *m/z* 255.13896 (C_17_H_19_O_2_^-^, Δmmu=−0.298); *m/z* 245.15460 (C_16_H_21_O_2_^-^, Δmmu=−0.103), *m/z* 215.14396 (C_15_H_19_O^-^, Δmmu=−0.179), *m/z* 177.09214 (C_11_H_13_O^-^, Δmmu=0.037) and *m/z* 137.09735 (C_9_H_13_O^-^, Δmmu=0.162). The loss of H_2_O (18 u) that yields *m/z* 255 is rather confusing since this type of neutral loss is not very frequent in negative even-electron ions [38]. However, loss of H_2_O was interpreted as the result of the formation of an alkynyl group after rupture of the C-OH bond at C-3 in OTP-318. A similar type of mechanism has been proposed during the fragmentation of ethers [41]. Observation of MS^3^ product ion scan spectrum of the E1-d_4_ ozonated SPE extract showed the presence of two ions which could be explained by this type of loss: *m/z* 259.16380 (C_17_H_15_D_4_O_2_^-^, Δmmu=0.737) and *m/z* 258.15754 (C_17_H_16_D_3_O_2_-, Δmmu=−0.343). The elemental composition of these two MS^3^ products ions thus seems to indicate that the loss of H_2_O or HDO is the result of the formation of a hydroxide anion complex.

The rest of the product ions do not indicate clearly the presence of other functional groups in the OTP-318 structure. The MS^4^ product ion scan (*m/z* 317 → *m/z* 273 → *m/z* 255 *↗ m/z* 75–350) yielded four main ions: *m/z* 240.11510 (C_16_H_16_O_2_^-^, Δmmu=−0.478), *m/z* 237.12804 (C17H17O-, Δmmu=−0.499), *m/z* 199.11260 (C_14_H_15_O^-^, Δmmu=−0.288) and *m/z* 159.08142 (C_11_H_11_O^-^, Δmmu=−0.118). From these the ion at *m/z* 237 suggested a second loss of H_2_O but it is not clear from which O atom (ketone group at C-10 or C-17) this loss was originated. As it was the case with OTP-276, the presence of *m/z* 240 in the MS^4^ product ion scan points towards the formation of an odd-electron ion, most likely from the loss of ^•^CH_3_ from C-13.

**Figure 3:**
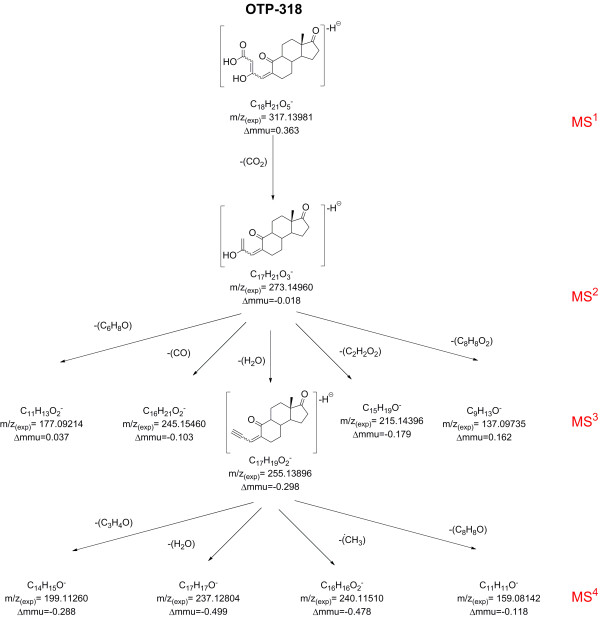
**Fragmentation tree of OTP-318 showing the most probable elemental composition of the main MS**^**n **^**product ions.**

In summary, MS^n^ experiments with the ozonated extracts of E1 and E1-d_4_ showed that a carboxylic acid and a hydroxyl group are present in the OTP-318 molecule. The other fragments showed in Figure 3 did not indicate unambiguously the presence of other functional groups in the molecular structure of OTP-318. Similarly to the fragments observed during the MS^n^ experiments performed with OTP-276, the elemental composition of the MS^3^ and MS^4^ product ions of OTP-318 pointed towards a partial preservation of the ring structure of E1. However, typical negative even-electron losses such as H_2_ and CH_4_ were observed less frequently than in OTP-276, probably due to the presence of 2 more O atoms in the structure OTP-318 parent ion which could have an impact on the possible fragmentation mechanisms. The proposed structures in Figure 1 (OTP-318A and OTP-318B) also hint that at least 4 configuration isomers are probable for this molecule given the presence of two carbon double bonds in its structure. This could explain the observation of at least three peaks at *m/z* 317.14 ± 0.01 in the chromatogram of the SPE extracts of the ozonated E1 solutions (Additional file 1: Figure S1).

## Conclusions

The present work showed that the identification, structural elucidation and confirmation of OTPs of contaminants of emerging concern can be significantly simplified using a control compare trend experiment followed by differential analysis and MS^n^ experiments. Application of this method to the study of the OTPs of E1 allowed the identification of two compounds of mass 276 u (OTP-276) and 318 u (OTP-318). Structural elucidation of these compounds was performed by MS^n^ (*n*=2-4) experiments via direct infusion into the mass spectrometer of SPE extracts of ozonated solutions of E1 and E1-d_4._ Observation of a neutral loss corresponding to CO_2_ in the MS^2^ product ion scan indicated the presence of a carboxylic acid group in the molecular structure of both OTPs. Also a loss of H_2_O observed in the MS^3^ product ion scan of OTP-318 suggested the presence of a hydroxyl group. The presence of these functional groups is consistent with known reaction mechanisms of O_3_[14,35] and previously suggested OTPs of hormones [11,12,15,27]. A study by de Oliveira Pereira, et al. [14] also identified OTP-276 as a main by-product of the ozonation of E1. However de Oliveira Pereira, et al. [14] and Huber, et al. [11] also found other OTPs that were not identified in the samples using the method presented in this paper. This might be a consequence of the different ozonation setups and experimental conditions used during the ozonation process.

While the method proposed here might oversight minor OTPs, it proved to be successful to eliminate most of the background noise, sample contaminants and signal spikes that are present in the acquisition files and to streamline the identification of the main OTPs. Removal of all the irrelevant data reduced to a great extent the amount of information that had to be processed manually (from 593 to 16 frames). Future work will focus on the optimization of software parameters to limit the number of candidate frames and reduce false positives. This method can now be more widely applied to the identification and elucidation of OTPs of other contaminants of emerging concern such as illicit drugs and antibiotics.

## Abbreviations

CECs: Contaminants of emerging concern; CID: Collision-induced dissociation; E1: Estrone; E1-d4: Deuterium-labeled estrone; GC-MS: Gas chromatography–mass spectrometry; HRMS: High-resolution mass spectrometry; LC-MS/MS: Liquid chromatography-tandem mass spectrometry; MSn: Multi-stage tandem mass spectrometry; NCE: Normalized collision energy; OTPs: Ozonation transformation products; RDBE: Ring and double bonds equivalents; TPs: Transformation products; tR: Retention time.

## Competing interests

The authors declare that they have no competing interests.

## Authors’ contributions

PAS participated in the conception and design of the experiments, in the acquisition, analysis and interpretation of data and in the drafting and revising of the manuscript. PK collaborated to the sample preparation and data analysis and also revised the manuscript. VY contributed to the design of the experiments, to the interpretation of the data and corrected the manuscript. All authors read and approved the final manuscript.

## Supplementary Material

Additional file 1: Figure S1 LC-HRMS chromatogram of the ozonated E1 preconcentrated sample. **Figure S2**. Fragmentation tree of OTP-279 (deuterated analogue of OTP-276). **Figure S3**. Fragmentation tree of OTP-322 (deuterated analogue of OTP-318). Click here for file
