# Supplementary material for: Identification and structural elucidation of ozonation transformation products of estrone
Source: Chem Cent J. 2013 Apr 23;7:74. doi: 10.1186/1752-153X-7-74 (PMC3643888; doi:10.1186/1752-153X-7-74)
Supplement: Additional file 1: Figure S1 — LC-HRMS chromatogram of the ozonated E1 preconcentrated sample. Figure S2. Fragmentation tree of OTP-279 (deuterated analogue of OTP-276). Figure S3. Fragmentation tree of OTP-322 (deuterated analogue of OTP-318). [file 1752-153X-7-74-S1.docx]

Identification and structural elucidation of transformation products of contaminants of estrone

Pedro A. Segura, Pearl Kaplan, Viviane Yargeau *

Department of Chemical Engineering, McGill University, 3610 University, Montreal, QC, H3A 2B2

* Corresponding author: viviane.yargeau@mcgill.ca

Tel: 1-514-398-2273, Fax: 1-514-398-6678

Additional Material


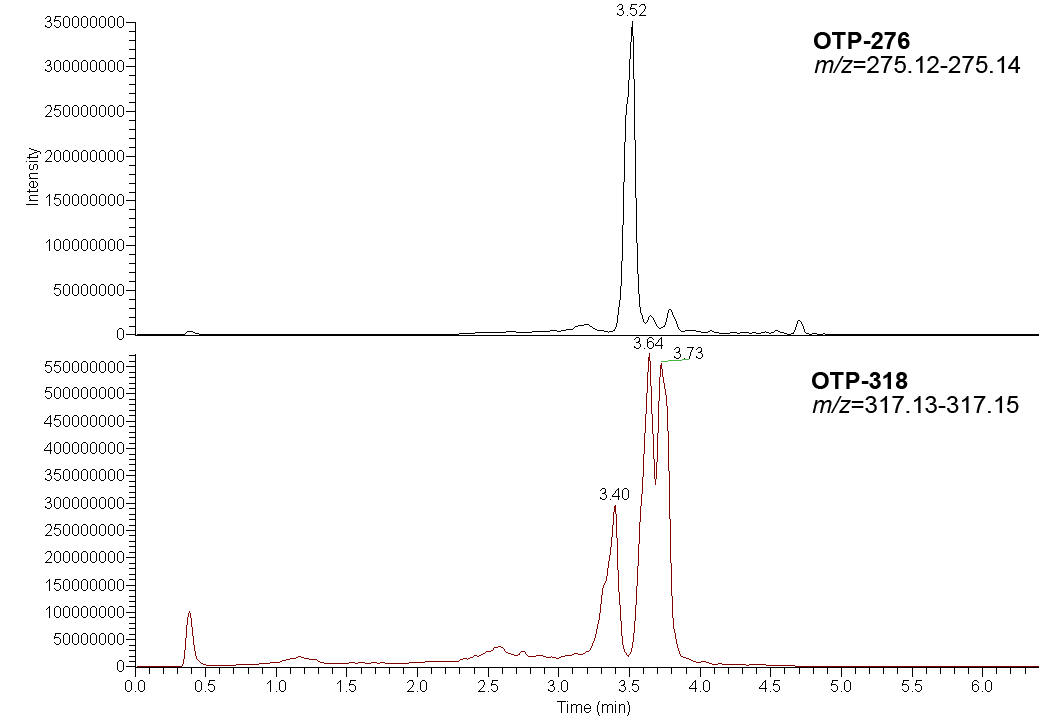


**Figure S1.** LC-HRMS chromatogram of the ozonated E1 preconcentrated sample. Signals for OTP-276 and OTP-318 were extracted with a mass window of ± 0.01 mmu.


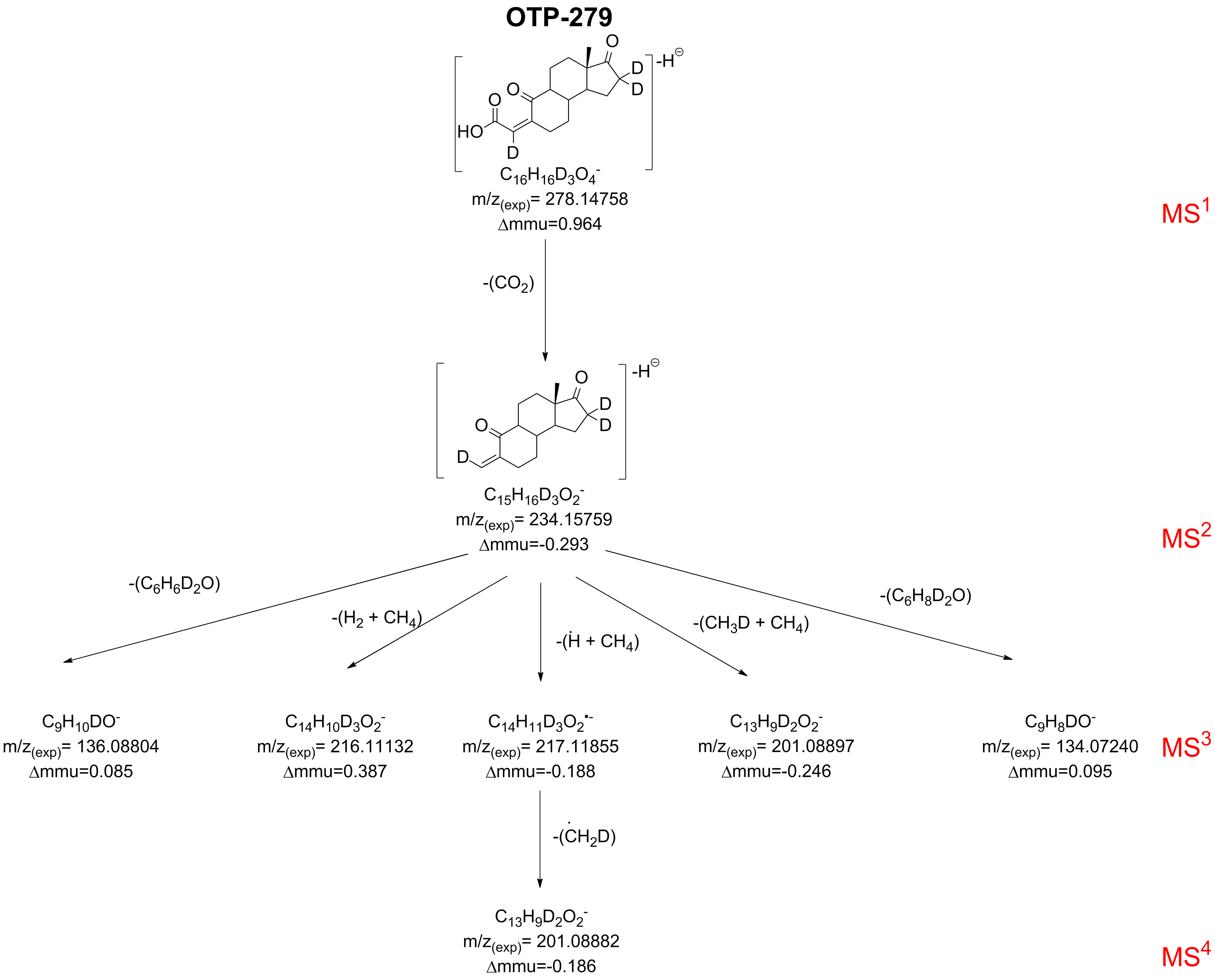


**Figure S2.** Fragmentation tree of OTP-279 (deuterated analogue of OTP-276) showing the most probable elemental composition of the main MS^n^ product ions.


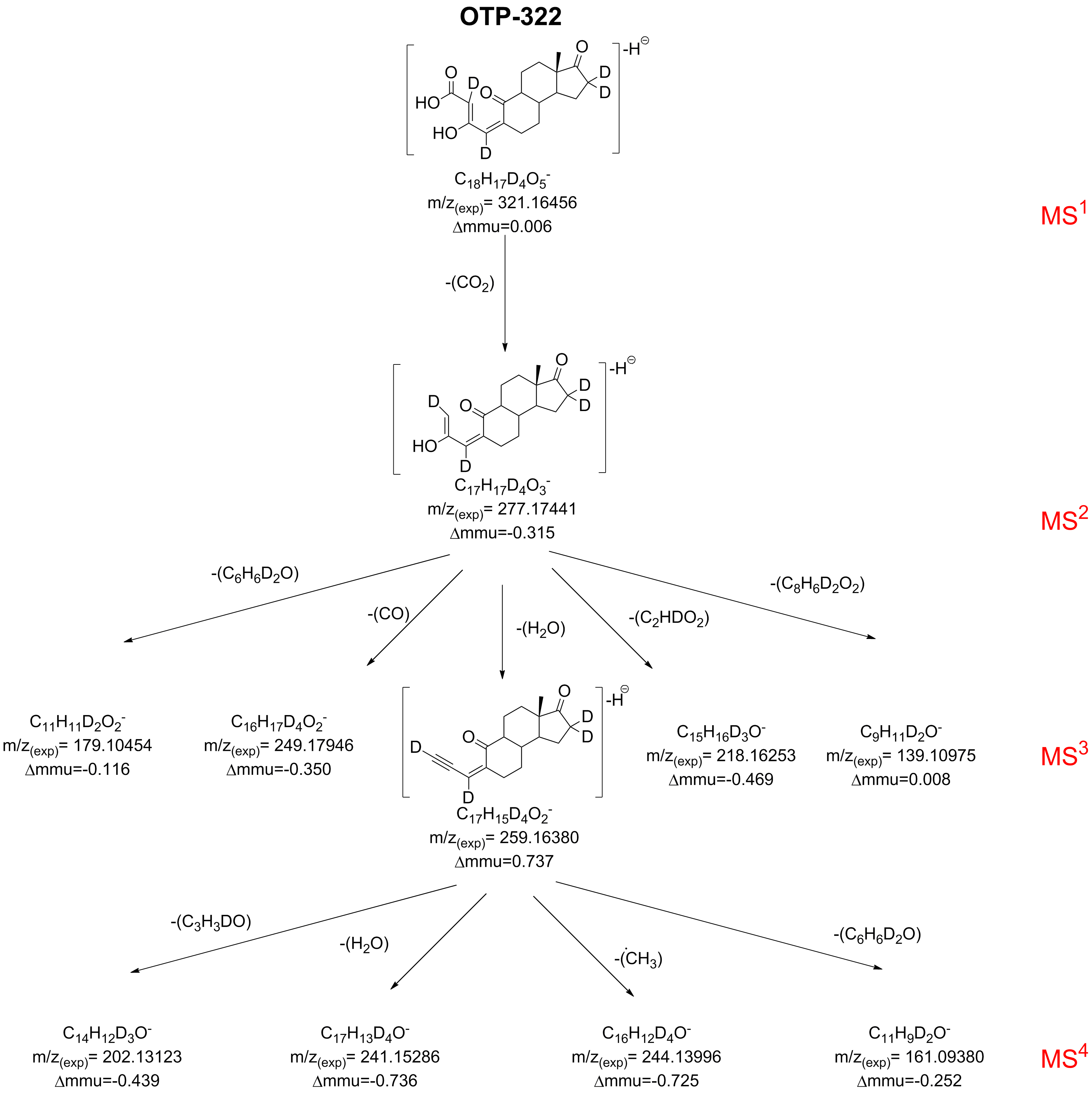


**Figure S3.** Fragmentation tree of OTP-322 (deuterated analogue of OTP-318) showing the most probable elemental composition of the main MS^n^ product ions.
